# Timely integration of palliative care. the reality check. a retrospective analysis

**DOI:** 10.1007/s00520-024-08721-x

**Published:** 2024-07-17

**Authors:** F. Adamidis, N. S. Baumgartner, A. Kitta, L. Kum, F Ecker, J Bär, C. Marosi, G. Kreye, C. Fischer, E. L. Zeilinger, C. Paschen, C. Wenzel, E. K. Masel

**Affiliations:** 1 Department of Medicine I, Division of Palliative Medicine, Medical University of Vienna, Vienna, Austria; 2 Department of Internal Medicine II, Clinical Division of Palliative Medicine, University Hospital Krems, Krems an Der Donau, Austria; 3 Karl Landsteiner University of Health Sciences, Krems an Der Donau, Austria; 4 Department of Health Economics, Center for Public Health, Medical University of Vienna, 1090 Vienna, Austria; 5 Department of Medicine III, Division of Nephrology and Dialysis, Medical University of Vienna, Vienna, Austria; 6 Department of Clinical and Health Psychology, Faculty of Psychology, University of Vienna, Vienna, Austria; 7 Department of Clinical Research SBG, Academy for Ageing Research, Haus Der Barmherzigkeit, Vienna, Austria

**Keywords:** Death, Neoplasms, Palliative Care, Public Health, Consultation and Referral, Terminal Care

## Abstract

**Purpose:**

A large volume of literature suggests that timely integration of palliative care (PC) enhances the well-being, quality of life and satisfaction of patients and their families. It may also positively impact clinical outcomes and healthcare costs throughout the disease trajectory. Therefore, reviewing clinical practice to reflect real-life situations regarding timely PC integration is essential.

**Methods:**

This study, conducted at the Vienna General Hospital between March 2016 and August 2022, retrospectively examined PC consultation (PCC) requests. It aimed to assess the timeliness of PC integration by analysing the duration between diagnosis and the first PCC request, as well as the interval between the first PCC request and death.

**Results:**

This study included 895 PCCs. The median time from diagnosis to the first PCC was 16.6 (interquartile range (IQR): 3.9–48.4) months, while the median time from the first PCC to death was 17.2 (IQR: 6.1–50.7) days. The median time from diagnosis to first PCC was 10.4 months in females (confidence interval (CI): 6.0–14.8) compared to 10.6 months in males (CI: 8.1–13.1; *p* = 0.675). There were no gender disparities in the time from first PCC to death, with a median of 23.3 days (CI: 15.6–31.0) for females and 22.3 days (CI: 16.2–28.4) for males (*p*  = 0.93). Fifty percent of patients died between 5 and 47 days after the first PCC.

**Conclusion:**

These findings highlight the discrepancy between the clinical perception of PC as end-of-life care and the existing literature, thereby emphasising the importance of timely PC integration.

**Supplementary Information:**

The online version contains supplementary material available at 10.1007/s00520-024-08721-x.

## Introduction

Palliative care (PC) is the active, holistic care of people of all ages with serious health-related distress [1]. PC is multifaceted and includes regular assessment of symptoms, education about the illness and prognosis, support in making treatment decisions and setting care goals, information about social support services, involvement of family caregivers and advance care planning. In the early stages of potentially life-threatening illnesses, many patients endure debilitating symptoms and psychosocial challenges, emphasising the need for timely PC integration [2].

Over the past decade, a substantial body of research has supported the incorporation of PC into the field of oncology for patients living with advanced cancer [3–5]. The focus has shifted from debating the need for PC to ascertaining the most effective approach to its implementation [6]. Key considerations now include determining the best delivery model, the optimal timing for referrals, the patients who would benefit the most from PC and the scope of PC responsibilities within the oncology community. Given the distressing symptoms and complexities encountered in the early treatment stages of serious illnesses, it is essential to identify the most effective methods and appropriate timing for integrating PC into the overall care pathway [7].

Since the landmark study by Temel et al. in 2010 [8], which examined the impact of early PC from the time of diagnosis in advanced lung cancer, the concept of early PC integration has gained prominence. The study demonstrated that patients with stage IV lung cancer benefited from the early PC perspective by preserving their quality of life, maintaining their social environment, implementing their treatment and care preferences and avoiding futile therapy and high costs [9].

The shift from the phrasing *early integration* of PC to *timely integration* of PC reflects a more nuanced and patient-centred approach. Both terms emphasise the importance of incorporating PC into a patient’s treatment plan but differ in their implications [10, 11]. The term *early* implies that PC should be offered as soon as a life-limiting illness is diagnosed, often alongside curative or disease-modifying treatments. By contrast, the term *timely* reflects a more flexible and individualised approach, recognising that the optimal timing for introducing PC should be determined by the patient’s individual circumstances and may evolve as the disease progresses [11]. This approach aligns with the principles of patient-centred care, ensuring that PC is integrated when it is most beneficial and supportive for the patient and their family.

In their mixed-methods study, Zimmermann et al. proposed the notion of the PC team as a proactive resource, emphasising its effectiveness in optimising efficiency within the healthcare system [12].

Many well-designed studies have shown that incorporating PC into treatment is beneficial and improves quality of life without reducing survival [1, 8, 13–18]. However, structured and timely integration of PC remains the exception rather than the norm.

General PC is defined as an approach that should be provided by healthcare professionals regardless of their speciality. Often referred to as *general* or *primary* PC, it requires routine assessment of symptoms, expertise in symptom management and communication skills. These communication skills include a willingness to discuss patients’ fears, concerns and end-of-life (EOL) issues without fear of destroying hope.

Specialist PC is provided by dedicated teams and applies to patients with both oncological and non-oncological conditions [19]. These teams should be involved in patient care based on the availability of services and the specific needs of the patients.

The European Society of Medical Oncology (ESMO) guidelines recommend that PC services should be evidence-based, integrated, dynamic and personalised. Ideally, PC should begin at the time of diagnosis and continue through survivorship or EOL [20]. ESMO also emphasises that PC should be provided alongside disease-modifying treatments and should not be limited to EOL care. The American Society of Clinical Oncology (ASCO) also provides guidelines for integrating PC into standard oncology care [15]. Furthermore, data indicate significant distress levels at the time of diagnosis of any serious illness, highlighting the need for early intervention as the disease progresses [21].

However, the implementation of timely PC involvement requires further refinement. The optimal timing for PC involvement depends on a combination of factors, including the patient’s burden and needs (physical symptoms, psychosocial burden, caregiver burden and prognostic aspects) and the goals that PC aims to achieve [22].

There is still no consensus on the best time to offer a PC consultation (PCC) during the course of the disease [23]. The present study is the first to examine a real-world PCC service in one of the largest academic hospitals in Europe. It aims to assess PC integration by analysing two timeframes: the period between diagnosis and the first request for PC and the interval between the first request for PC and death. These assessments provide insights into the timeliness of PC initiation after diagnosis and the duration of PC support before death, thereby improving our understanding of the effectiveness of PC integration and its impact on patient care and outcomes.

## Methods

### Data acquisition

The study sample includes cases of consultation requests from wards seeking PC within their own setting, as well as cases where transfer to the PC ward is deemed necessary. It involves patients who received PCC services between March 2016 and August 2022 at Vienna General Hospital, the largest academic hospital in Austria and one of the largest hospitals in Europe. Patients with insufficient documentation of their consultations were excluded. Digital patient records were reviewed using the hospital’s computer information management system (AKIM). Demographic information, including age, sex, primary diagnosis and PCC-related data, was extracted for each participant. Only authorised personnel had access to the original data. Prior to analysis, study-relevant data were pseudonymised using a sequential pseudonymisation number, ensuring that only authorised personnel had access to the original data.

The study was conducted in accordance with the Declaration of Helsinki and was approved by the Ethics Committee of the Medical University of Vienna (EK Number: 1333/2019).

### PCC service at Vienna General Hospital

The Vienna General Hospital has approximately 1,700 beds with an integrated comprehensive cancer centre and a 12-bed PC unit (PCU) providing specialised PC. The PCU records approximately 350 admissions per year. The admission process to the PCU involves three pathways: 1) *PCC Service*: patients are assessed by a multidisciplinary team, including a PC physician, to determine their needs and suitability for inpatient care; 2) *Outpatient Clinic for Palliative Medicine*: patients already receiving outpatient PC may be referred for inpatient admission due to worsening symptoms or complex needs; and 3) *Referral by Mobile PC Teams or Primary Care Physicians*: mobile PC teams or attending physicians can initiate admission by telephone for patients requiring inpatient support.

A team consisting of a doctor and a nurse from the PCC service provide PCCs to various units at the Vienna General Hospital. The attending clinician must complete a consultation order form prior to the consultation, specifying whether the patient should be referred to the PCU or if the requesting unit only needs a consultation with the PC team. Possible reasons for requesting a consultation (multiple responses are possible) include pain, shortness of breath, nausea/vomiting, psychological problems, nutritional problems, social situation, carer relief, terminal care and care problems. The level of care required by each patient is also assessed. It is important to note whether a social worker has been involved previously and whether the patient has been informed about their current medical condition and the upcoming visit by the PC team. The present study focused on the first contact between a patient and the PC team, excluding PCU admissions without prior PCC.

### Statistical analysis

Data were reported as median, interquartile range (IQR) and total range. Cumulative incidences of events were examined using the log-rank test and presented using Kaplan–Meier plots. The endpoints were survival since the diagnosis of a life-limiting disease, time from diagnosis until the first PCC and time from the first PCC to death. The statistical analysis was conducted in two stages. First, the focus was on the most frequent tumour groups (lung cancer, pancreatic cancer, head and neck cancer, and colorectal cancer). Second, these tumour groups were examined by sex, excluding sex-specific diseases like breast and prostate cancers. A two-sided p-value < 0.05 was considered significant. Statistical analysis was performed using SPSS (v.29) and GraphPad Prism (v.8.0.1).

## Results

### Patient characteristics

The total sample consisted of 935 patients (50.9% female). The analysis included 895 patients who received a PCC and subsequently died (Table 1). The median age at diagnosis of the life-limiting illness was 64.7 (IQR: 54.0–73.5) years, ranging from 16.2 to 97.0 years. The median age at death was 67.8 (IQR: 57.3–76.5) years, ranging from 18.4 to 97.1 years. The median survival from diagnosis was 18.4 months, with an IQR of 5.7 to 50.8 months. Patients spent a median of 20.3 (IQR: 10.1–36.5) days in the hospital.

**Table 1:** Demographic and clinical characteristics of patients who received a palliative care consultation (PCC) (*n* = 935)

Female – no. (%)	476	(50.9%)
Dead – no. (%)	40	(95.7%)
Age at diagnosis – years	64.7	(54.0–73.5)
Age at death – years	67.8	(57.3–76.5)
Survival since life-limiting diagnosis – months	18.4	(5.7–50.8)
Time to PCC – months	16.6	(3.9–48.4)
Time from PCC to death – days	17.2	(6.1–50.7)
Relative time from PCC to death related to survival – %	3.5	(0.9–15.5)
Lung cancer – no. (%)	131	(14%)
Pancreatic cancer – no. (%)	67	(7.2%)
Colorectal cancer – no. (%)	56	(6.0%)
Head and neck cancer – no. (%)	76	(8.1%)
Breast cancer – no. (%)	72	(7.7%)
Prostate cancer – no. (%)	32	(3.4%)

Legend: Metric data are displayed as median and interquartile range. *PC* palliative care

**Fig. 1:**
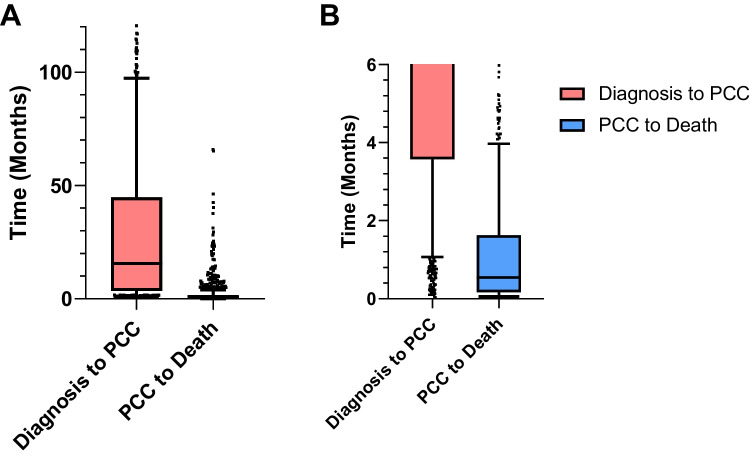
Comparison of time elapsed from life-limiting diagnosis to first palliative care consultation (PCC) and time from PCC to death. **A** The entire range of time. **B** Zoomed-in view of the box plot for the time from PCC to death. Values are displayed as medians and IQRs

Regarding PCCs, the median time from diagnosis was 16.6 (IQR: 3.9–48.4) months, while the median time from the first PCC to death was 17.2 (IQR: 6.1–50.7) days (Fig. 1). The total time from consultation to death ranged from zero days to 5.4 years. A total of 19 patients (2.1%) died on the day of their first PCC.

### Analysis of frequent life-limiting diagnosis

**Fig. 2:**
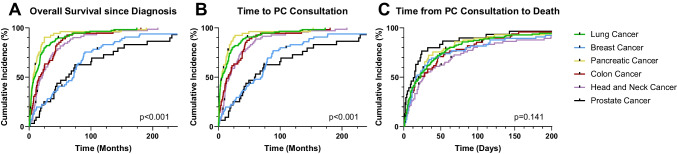
Association of the most frequent oncologic diagnosis and overall survival since life-limiting diagnosis (**A**), time from life-limiting diagnosis and first PCC (**B**) and time from PCC to death (**C**)

The most common life-limiting diagnoses in the cohort (*n* = 434; 51.4% female; Table 2) were lung cancer (*n*  = 131; 14.0%), head and neck cancer (*n*  = 76; 8.1%), breast cancer (*n*  = 72; 7.7%), pancreatic cancer (*n*  = 67; 7.2%), colorectal cancer (*n*  = 56; 6.0%) and prostate cancer (*n*  = 32; 3.4%). The earliest diagnosis of a life-limiting condition occurred at a median age of 56.1 (CI: 52.2–60.1) years for breast cancer (*p* = 0.01). Median survival was shortest for pancreatic cancer (6.6 [CI: 3.7–9.5] months) and lung cancer (8.6 [CI: 4.1–13.1] months) and longest for breast cancer (72.0 (CI: 59.2–84.8) months) (*p* < 0.001; Fig. 2A). The time from the primary diagnosis to the first PCC varied from less than one year up to ≥ 5 years (*p*  < 0.001; Fig. 2B). The median time to PCC was 5.2 (CI: 3.0–7.4) months for pancreatic cancer and 6.6 (CI: 2.3–10.9) months for lung cancer. For breast cancer, the median time was 70.2 (CI: 48.0–92.4) months. There were no apparent diagnosis-related differences in the time from the first PCC to death (*p* = 0.141; Fig. 2C).

**Table 2:** Clinical characteristics of the most frequent oncologic diseases

	Age at diagnosis (Years)		Age at death (Years)		Survival since diagnosis (Months)		Time to PCC (Months)		Time from PCC to death (Days)		Relative time from PCC to death related to survival (%)	
	*Median*	*CI*	*Median*	*CI*	*Median*	*CI*	*Median*	*CI*	*Median*	*CI*	*Median*	*CI*
Lung Cancer (*n* = 131)	67.2	65.2–69.3	68.4	66.3–70.5	8.57	4.1–13.1	6.6	2.3–10.9	21.3	13.6–29.0	8.5	2.5–14.5
Pancreatic Cancer (*n* = 67)	67.3	63.0–71.6	69.7	60.0–69.8	6.6	3.7–9.5	5.2	3.0–7.4	21.3	11.7–30.9	19.0	6.7–31.4
Colorectal Cancer (*n* = 56)	62.5	57.2–67.8	65.3	61.9–68.7	16.0	6.3–25.6	15.6	6.3–24.9	17.2	1.1–33.4	4.6	2.2–7.1
Head and Neck Cancer (*n* = 76)	62.8	57.7–67.9	67.0	63.0–71.0	21.1	14.6–27.6	17.7	10.0–25.4	26.4	16.0–36.8	6.0	3.2–8.9
Breast Cancer (*n* = 72)	56.1	52.2–60.1	64.9	60.0–69.8	72.0	59.2–84.8	70.2	48.0–92.4	17.2	9.7–24.8	2.4	1.7–3.1
Prostate Cancer (*n* = 32)	67.4	62.6–72.3	75.3	72.7–77.9	59.6	27.4–91.9	58.5	26.6–90.5	13.2	5.0–21.4	0.7	0.2–1.2
p-value	0.01		0.09		< 0.001		< 0.001		0.141		< 0.001	
Frequent Diagnosis (Lung Cancer, Pancreatic Cancer, Colorectal Cancer, Head and Neck Cancer)												
Female (*n* = 151)	67.4	64.1–70.7	68.9	66.3–71.5	11.4	7.9–14.8	10.4	6.0–14.8	23.3	15.6–31.0	10.9	5.3–16.6
Male (*n* = 179)	65.2	62.7–67.7	66.9	64.5–69.4	13.1	10.4–15.8	10.6	8.1–13.1	22.3	16.2–28.4	6.0	4.6–7.5
p-value	0.074		0.057		0.627		0.675		0.93		0.044	

Legend: PC: Palliative Care

The longest time from PCC to death was observed in patients with head and neck cancer (26.4 [CI: 16.0–36.8] days), and the shortest time was 13.2 (CI: 5.0–21.4) days in patients with prostate cancer. The highest observed percentage of time from PCC to death relative to the time since diagnosis was 19.0% (6.7–31.4) in pancreatic cancer, while the lowest percentage was 0.7% (0.2–1.2) in prostate cancer (*p* < 0.001). Further time-related data according to life-limiting diagnosis are shown in Table 2.

### Disparities between sexes

In the general cohort, the median time from diagnosis to death was 21.1 (CI: 17.5–24.7) months for females and 16.7 (CI: 14.4–19.0) months for males (*p* = 0.027; Supplemental Fig. 1). The time from life-limiting diagnosis to the first PCC was 19.2 (CI: 16.0–22.4) months for females compared to 15.0 (CI: 11.9–18.1) months for males (*p* = 0.032). Female patients died within 19.2 (CI: 15.8–22.7) days after the first PCC, while male patients died within 16.2 (CI: 13.4–19.1) days (*p* = 0.123). The relative time from PCC to death during the period from diagnosis to death was 3.8% (CI: 2.9–4.6) in females and 4.0% (CI: 3.0–5.0) in males (*p* = 0.518).

**Fig. 3:**
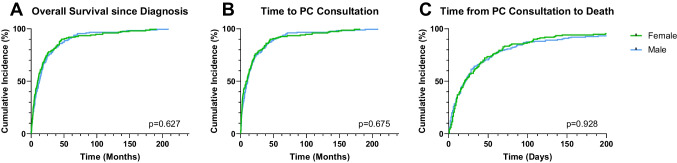
Association of patient’s sex in the isolated cohort with the most frequent oncologic diagnosis and overall survival since life-limiting diagnosis (**A**), time from life-limiting diagnosis and first PCC (**B**) and time from PCC to death (**C**)

Focusing on the frequent diagnosis (lung cancer, pancreatic cancer, colorectal cancer and head and neck cancer; *n*  = 330; 45.8% female) and excluding sex-specific diseases (breast and prostate cancers), the median age at the diagnosis of a life-limiting illness was 67.4 years for females (CI: 64.1–70.7 years) and 65.2 years for males (CI: 62.7–67.7 years; *p* = 0.074). Median survival from primary diagnosis was 11.4 (CI: 7.9–14.8) months for females and 13.1 (CI: 10.4–15.8) months for males (*p* = 0.627; Fig. 3A). The median time from diagnosis to the first PCC was 10.4 months for females (CI: 6.0–14.8 months) and 10.6 months for males (CI: 8.1–13.1 months), with no statistically significant difference (*p* = 0.675; Fig. 3B). There were no sex differences in the time elapsed from the first PCC to the time of death, with a median of 23.3 (CI: 15.6–31.0) days for females and 22.3 (CI: 16.2–28.4) days for males (*p* = 0.928; Fig. 3C). The relative time from the first PCC to death during the remaining time since diagnosis to death was 10.9% (CI: 5.3–16.6) in females and 6.0% (CI: 4.6–7.5) in males (*p* = 0.044).

## Discussion

The results of this study underscore a prevailing pattern where timely engagement with PC remains rare among patients with a life-limiting diagnosis. Instead, the majority of patients were referred for a PCC during the EOL phase or even at EOL itself. The results suggest that the enduring perception of PC is still primarily associated with the care of the dying. This perspective is exemplified by the observation that out of 935 patients referred for PCC, a substantial 895 died within a median of 17.2 days after their first PCC. Over 75% of patients referred for PCC died within less than 51 days. This pattern was consistent across common diagnoses and regardless of sex.

Consistent intervals between PCC and time of death were observed regardless of whether the prognosis was unfavourable, as in advanced lung and pancreatic cancers, or suggested the possibility of prolonged survival, as in prostate and breast cancers. The percentage of time from PCC to death, relative to the time from diagnosis to death, was higher in cancers with unfavourable outcomes (e.g. pancreatic cancer).

We observed sex differences within the general cohort, where the survival since diagnosis and time to PCC was longer for women. Interestingly, no such difference was observed in the time from PCC to death and the relative time from PCC to death. This observation led us to conclude that PCCs were sought at a similar time just before death. Examining sex differences in the most common diseases in our dataset, we found that the time from PCC to death and from diagnosis to PCC showed parallel patterns for both female and male patients. Notably, the percentage of time from PCC to death regarding the remaining lifetime since diagnosis was higher in female patients.

A systematic review by Bennardi and colleagues identified significant barriers to the integration of PC in haemato-oncology patients [24]. Among these barriers are the lack of awareness, experience and training of healthcare providers in the principles and practice of PC, as well as uncertainty about the optimal timing for PC interventions. An international consensus process identified patient-related triggers for timely PC, such as high symptom burden, high emotional distress, desire to die, prognosis of 6–12 months, progression after first-line palliative therapy and an Eastern Cooperative Oncology Group (ECOG) stage 2 status [25]. In a consensus process by Hui et al., there was no clear vote for either an ‘automatic’ inclusion (e.g. from a certain tumour stage) or a ‘referral-based’ inclusion, which would be decided individually; instead, a mixture of both approaches was recommended [26]. Ideally, PC should be initiated as early as possible in the diagnosis of an advanced incurable disease. It can be provided alongside disease-specific therapy to tailor PC to the individual patient and their needs and can also be initiated after the end of disease-specific therapy. The cornerstones of PC are the realistic achievement of defined treatment goals, advanced care planning, symptom management and ensuring that therapeutic benefits outweigh potential harms. In the coming years, it will be crucial to regularly identify individuals with complex healthcare needs and significant symptom-related challenges, as they are likely to benefit most from the timely initiation of specialised PC [27]. These studies suggest a paradigm shift: the timing of PC should be based on individual needs rather than prognostic considerations.

PC can reduce anxiety and improve psychosocial symptom burden by providing prognostic information [28]. Aggressive treatments, prolonged hospital stays and emergency department visits may indicate poor quality of life at the EOL. Ideally, this can be prevented by timely PC [29]. Despite the scientific evidence, the practical application of timely PC is still scarce. Given our ageing society and ongoing medical advances, where many diseases are no longer curable but offer an improved prognosis, distressing symptoms should be identified as early as possible.

The results of the current study underline that PCCs should not only be offered to dying patients in their last days but should also provide targeted care and holistic symptom relief early in the course of the disease. Additionally, timely PC could help patients cope with their illness and prepare for the future. Timely PC could also reduce the number of deaths in hospitals. If patients are referred for PC shortly before death, there may be less time to respect patients’ individual goals and values. The frequent demand for PC skills should indicate that routine PC training should be available across different medical specialities to improve understanding and appreciation of the role of a PC team [30].

### Strengths and limitations

When interpreting the results of the current study, it is important to consider its retrospective nature. Notably, this study focuses on PCC at a single centre. However, the aim was to determine the actual referral patterns for timely PC in the largest hospital in Vienna.

Although Vienna General Hospital is the largest hospital in the country and one of the largest hospitals in Europe, our data cannot be considered representative of the entire country due to varying PC structures in different federal states. Furthermore, we cannot exclude the possibility that patients may have had contact with a PC team outside the hospital, although this is unlikely based on our experience.

### Practical implications

Timely PC should not necessarily be used synonymously with timely specialist PC, as much of PC is provided as basic oncology PC. For the integration of specialist PC, identifying triggers for timely PC is necessary to facilitate meaningful and effective integration. Such collaborations should be based on patient needs and consider availability and resources. PCCs require human resources, making the availability of PC services crucial in determining the best place of care in consultation with patients, their caregivers and the medical team. The timely integration of PC services into Comprehensive Cancer Centres is essential. Additionally, providing PC to patients with non-malignant conditions remains a challenge [31]. Multi-professional and multi-sectoral comprehensive care is a major challenge. Quality control and patient-reported outcome measures are essential to assess the type of care provided by different PC services, evaluate treatment outcomes and promote early PC based on outcome data [16, 32]. Prognostic management approaches include recommending PC support at the time of diagnosis of stage IV disease or using the surprise question: ‘Would you be surprised if the patient died within the next year?’ [33, 34]. The results of a randomised controlled trial in patients with advanced cancer patients show that early and systematic integration of PC is more beneficial than PCCs offered on an as-needed basis [35]. Regular assessment of symptoms can lead to significant improvements in quality of life and even overall survival [23]. The goal of timely integration of PC could be achieved through low-threshold services, such as outpatient services, 24-h telephone numbers and the availability of primary PC services. Such a low-threshold approach may also be appropriate for patients with a longer life expectancy and less aggressive disease. For PC to be effective at an early stage, PC providers should also be open to concurrent disease-related therapies. Health professionals in different settings need to reflect on their own attitudes towards PC. A large number of studies [25–27] have demonstrated the positive effects of timely PC on various outcome parameters later in the course of the disease using very different approaches. Dual awareness is needed to meet the requirements of both modern disease-oriented treatment concepts and comprehensive PC.

## Conclusion

The findings of this study highlight the ongoing challenges associated with providing timely PC in the real-world setting of a tertiary care centre. In summary, the results emphasise the critical need for enhanced education regarding the importance of timely PC. The scarcity of resources available for PC and the prevailing misconception of PC as primarily EOL care underscore the urgency of PC education.

Identification of triggers for timely PC integration is essential to facilitate maximum benefit. The implementation of PC should be based on the needs of the patient while considering availability and resource constraints.

Timely PC can significantly impact the prioritisation of care goals, which may include improving quality of life, enhancing communication, increasing prognostic awareness and providing support for family caregivers.

## Supplementary Information

Below is the link to the electronic supplementary material.

Supplementary file1 (DOCX 28 KB)

## Data Availability

No datasets were generated or analysed during the current study.

## References

[1] Vanbutsele G, Pardon K, Van Belle S et al (2018) Effect of early and systematic integration of palliative care in patients with advanced cancer: a randomised controlled trial. Lancet Oncol 19:394–404. 10.1016/S1470-2045(18)30060-3 29402701 10.1016/S1470-2045(18)30060-3

[2] Kaasa S, Loge JH, Aapro M et al (2018) Integration of oncology and palliative care: a Lancet Oncology Commission. Lancet Oncol 19:e588–e653. 10.1016/S1470-2045(18)30415-7 30344075 10.1016/S1470-2045(18)30415-7

[3] Maltoni M, Scarpi E, Dall’Agata M, et al (2016) Systematic versus on-demand early palliative care: A randomised clinical trial assessing quality of care and treatment aggressiveness near the end of life. Eur J Cancer 69:110–118. 10.1016/j.ejca.2016.10.004 27821313 10.1016/j.ejca.2016.10.004

[4] Scarpi E, Dall’Agata M, Zagonel V, et al (2019) Systematic vs. on-demand early palliative care in gastric cancer patients: a randomized clinical trial assessing patient and healthcare service outcomes. Support Care Cancer 27:2425–2434. 10.1007/s00520-018-4517-2 30357555 10.1007/s00520-018-4517-2

[5] Temel JS, Sloan J, Zemla T et al (2020) Multisite, Randomized Trial of Early Integrated Palliative and Oncology Care in Patients with Advanced Lung and Gastrointestinal Cancer: Alliance A221303. J Palliat Med 23:922–929. 10.1089/jpm.2019.0377 32031887 10.1089/jpm.2019.0377 PMC7307668

[6] Hui D, Hannon BL, Zimmermann C, Bruera E (2018) Improving patient and caregiver outcomes in oncology: Team-based, timely, and targeted palliative care. CA Cancer J Clin 68:356–376. 10.3322/caac.21490 30277572 10.3322/caac.21490 PMC6179926

[7] Zimmermann C, Swami N, Krzyzanowska M et al (2014) Early palliative care for patients with advanced cancer: a cluster-randomised controlled trial. Lancet 383:1721–1730. 10.1016/S0140-6736(13)62416-2 24559581 10.1016/S0140-6736(13)62416-2

[8] Temel JS, Greer JA, Muzikansky A et al (2010) Early palliative care for patients with metastatic non-small-cell lung cancer. N Engl J Med 363:733–742. 10.1056/NEJMoa1000678 20818875 10.1056/NEJMoa1000678

[9] Smith S, Brick A, O’Hara S, Normand C (2014) Evidence on the cost and cost-effectiveness of palliative care: a literature review. Palliat Med 28:130–150. 10.1177/0269216313493466 23838378 10.1177/0269216313493466

[10] Schenker Y, Arnold R (2017) Toward Palliative Care for All Patients With Advanced Cancer. JAMA Oncol 3:1459–1460. 10.1001/jamaoncol.2017.1059 28520825 10.1001/jamaoncol.2017.1059

[11] Hui D, Heung Y, Bruera E (2022) Timely Palliative Care: Personalizing the Process of Referral. Cancers (Basel) 14:1047. 10.3390/cancers14041047 35205793 10.3390/cancers14041047 PMC8870673

[12] Zimmermann C, Pope A, Hannon B et al (2023) Symptom screening with Targeted Early Palliative care (STEP) versus usual care for patients with advanced cancer: a mixed methods study. Support Care Cancer 31:404. 10.1007/s00520-023-07870-9 37341839 10.1007/s00520-023-07870-9

[13] Radbruch L, De Lima L, Knaul F et al (2020) Redefining Palliative Care-A New Consensus-Based Definition. J Pain Symptom Manage 60:754–764. 10.1016/j.jpainsymman.2020.04.027 32387576 10.1016/j.jpainsymman.2020.04.027 PMC8096724

[14] McAteer R, Wellbery C (2013) Palliative care: benefits, barriers, and best practices. Am Fam Physician 88:807–813 24364543

[15] Ferrell BR, Temel JS, Temin S et al (2017) Integration of Palliative Care Into Standard Oncology Care: American Society of Clinical Oncology Clinical Practice Guideline Update. J Clin Oncol 35:96–112. 10.1200/JCO.2016.70.1474 28034065 10.1200/JCO.2016.70.1474

[16] Hoerger M, Greer JA, Jackson VA et al (2018) Defining the Elements of Early Palliative Care That Are Associated With Patient-Reported Outcomes and the Delivery of End-of-Life Care. J Clin Oncol 36:1096–1102. 10.1200/JCO.2017.75.6676 29474102 10.1200/JCO.2017.75.6676 PMC5891131

[17] Temel JS, Greer JA, El-Jawahri A et al (2017) Effects of Early Integrated Palliative Care in Patients With Lung and GI Cancer: A Randomized Clinical Trial. J Clin Oncol 35:834–841. 10.1200/JCO.2016.70.5046 28029308 10.1200/JCO.2016.70.5046 PMC5455686

[18] Temel JS, Petrillo LA, Greer JA (2022) Patient-Centered Palliative Care for Patients With Advanced Lung Cancer. J Clin Oncol 40:626–634. 10.1200/JCO.21.01710 34985932 10.1200/JCO.21.01710

[19] Periyakoil VS, von Gunten CF, Fischer S et al (2022) Generalist versus Specialist Palliative Medicine. J Palliat Med 25:193–199. 10.1089/jpm.2021.0644 35103529 10.1089/jpm.2021.0644 PMC9022124

[20] Jordan K, Aapro M, Kaasa S et al (2018) European Society for Medical Oncology (ESMO) position paper on supportive and palliative care. Ann Oncol 29:36–43. 10.1093/annonc/mdx757 29253069 10.1093/annonc/mdx757

[21] Fitzsimons D, Mullan D, Wilson JS et al (2007) The challenge of patients’ unmet palliative care needs in the final stages of chronic illness. Palliat Med 21:313–322. 10.1177/0269216307077711 17656408 10.1177/0269216307077711

[22] Kayastha N, LeBlanc TW (2020) When to Integrate Palliative Care in the Trajectory of Cancer Care. Curr Treat Options Oncol 21:41. 10.1007/s11864-020-00743-x 32328882 10.1007/s11864-020-00743-x

[23] Bakitas MA, Tosteson TD, Li Z et al (2015) Early Versus Delayed Initiation of Concurrent Palliative Oncology Care: Patient Outcomes in the ENABLE III Randomized Controlled Trial. J Clin Oncol 33:1438–1445. 10.1200/JCO.2014.58.6362 25800768 10.1200/JCO.2014.58.6362 PMC4404422

[24] Bennardi M, Diviani N, Gamondi C et al (2020) Palliative care utilization in oncology and hemato-oncology: a systematic review of cognitive barriers and facilitators from the perspective of healthcare professionals, adult patients, and their families. BMC Palliat Care 19:47. 10.1186/s12904-020-00556-7 32284064 10.1186/s12904-020-00556-7 PMC7155286

[25] Müller S, Fink M, Hense J et al (2022) Palliative care outpatients in a German comprehensive cancer center—identifying indicators for early and late referral. BMC Palliat Care 21:221. 10.1186/s12904-022-01114-z 36503625 10.1186/s12904-022-01114-z PMC9743520

[26] Hui D, Bruera E (2016) Integrating palliative care into the trajectory of cancer care. Nat Rev Clin Oncol 13:159–171. 10.1038/nrclinonc.2015.201 26598947 10.1038/nrclinonc.2015.201 PMC4772864

[27] Gärtner J, Alt-Epping B, Daun M (2018) Palliative Care - not just for the final phase. A rewiev of evidence. Ther Umsch 75:123–126. 10.1024/0040-5930/a000977 30022722 10.1024/0040-5930/a000977

[28] Enzinger AC, Zhang B, Schrag D, Prigerson HG (2015) Outcomes of Prognostic Disclosure: Associations With Prognostic Understanding, Distress, and Relationship With Physician Among Patients With Advanced Cancer. J Clin Oncol 33:3809–3816. 10.1200/JCO.2015.61.9239 26438121 10.1200/JCO.2015.61.9239 PMC4737862

[29] Hirvonen OM, Leskelä R-L, Grönholm L et al (2020) The impact of the duration of the palliative care period on cancer patients with regard to the use of hospital services and the place of death: a retrospective cohort study. BMC Palliat Care 19:37. 10.1186/s12904-020-00547-8 32209075 10.1186/s12904-020-00547-8 PMC7093948

[30] Frydman JL, Hauck K, Lowy J, Gelfman LP (2021) Improving the Care of Patients With Serious Illness: What Are the Palliative Care Education Needs of Internal Medicine Residents? Am J Hosp Palliat Care 38:1218–1224. 10.1177/1049909120987207 33478256 10.1177/1049909120987207 PMC9979276

[31] Traue DC, Ross JR (2005) Palliative care in non-malignant diseases. J R Soc Med 98:503–506 16260799 10.1258/jrsm.98.11.503 PMC1275998

[32] Anand S, Glaspy J, Roh L et al (2020) Establishing a Denominator for Palliative Care Quality Metrics for Patients with Advanced Cancer. J Palliat Med 23:1239–1242. 10.1089/jpm.2019.0346 31928372 10.1089/jpm.2019.0346

[33] Davis MP, Vanenkevort E (2022) The Surprise Question. BMJ Support Palliat Care 12:403–406. 10.1136/spcare-2022-003853 36038254 10.1136/spcare-2022-003853

[34] Yen Y-F, Lee Y-L, Hu H-Y et al (2022) Early palliative care: the surprise question and the palliative care screening tool-better together. BMJ Support Palliat Care 12:211–217. 10.1136/bmjspcare-2019-002116 32451326 10.1136/bmjspcare-2019-002116

[35] Vanbutsele G, Van Belle S, Surmont V et al (2020) The effect of early and systematic integration of palliative care in oncology on quality of life and health care use near the end of life: A randomised controlled trial. Eur J Cancer 124:186–193. 10.1016/j.ejca.2019.11.009 31812934 10.1016/j.ejca.2019.11.009

