# Supplementary material for: Timely integration of palliative care. the reality check. a retrospective analysis
Source: Support Care Cancer. 2024 Jul 17;32(8):518. doi: 10.1007/s00520-024-08721-x (PMC11254969; doi:10.1007/s00520-024-08721-x)
Supplement: Supplementary file 1 — Supplementary file1 (DOCX 28 KB) [file 520_2024_8721_MOESM1_ESM.docx]

**Supplemental Figure 1**: Association of Patient’s Sex and Lifetime (A), Overall Survival Since Life-limiting Diagnosis (B), Time from Life-limiting Diagnosis and first Palliative Care Consultation (C) and Time from PC consultation to Death (D).
